# Persistent Autonomic Engagement and Cardiac Control After Four or More Years of Autonomic Regulation Therapy Using Vagus Nerve Stimulation

**DOI:** 10.3389/fphys.2022.853617

**Published:** 2022-03-11

**Authors:** Imad Libbus, Rajendra K. Premchand, Kamal Sharma, Sanjay Mittal, Rufino Monteiro, Badri Amurthur, Bruce H. KenKnight, Lorenzo A. DiCarlo, Inder S. Anand

**Affiliations:** ^1^LivaNova USA, Inc., Houston, TX, United States; ^2^Krishna Institute of Medical Sciences, Secunderabad, India; ^3^Sanjivani Super Specialty Hospitals, Ahmedabad, India; ^4^Medanta, The Medicity, Haryana, India; ^5^Vintage Hospital, Panaji, India; ^6^Emeritus, University of Minnesota, Minneapolis, MN, United States

**Keywords:** heart failure, autonomic regulation therapy, vagus nerve stimulation, non-pharmacological therapy, guideline directed medical therapy

## Abstract

**Introduction:**

Although heart failure (HF) outcomes have improved dramatically with the use of guideline directed medical therapy and implantable devices, the overall prognosis of patients with HF and reduced ejection fraction (HFrEF) remains poor. Autonomic Regulation Therapy (ART) using chronic vagus nerve stimulation (VNS) has been evaluated in the ANTHEM-HF study, using changes in heart rate (HR) dynamics as a biomarker of autonomic nervous system engagement and cardiac control to guide VNS titration. ART was associated with sustained improvement in cardiac function and HF symptoms in patients with HFrEF and persistent HF symptoms despite guideline-directed medical therapy (GDMT). We sought to determine whether the responsiveness of the autonomic nervous system to ART, as reflected in HR response to vagus stimulation during the VNS duty cycle, is maintained after long-term chronic VNS administration.

**Methods:**

Fifteen patients with HFrEF and implanted with a VNS systems in the ANTHEM-HF study were evaluated after 4.7 ± 0.3 years (range: 4.0–5.0 years) of chronic ART. ECG electrodes were placed on each patient’s wrists, and ECG rhythm strips were recorded. Instantaneous HR time series was computed at each patient’s chronically programmed VNS intensity and during progressively increasing VNS intensity. HR during active stimulation (on-time) was compared to HR just prior to initiation of each stimulation cycle (off-time).

**Results:**

Persistent autonomic engagement was observed in a majority of patients (11 of 15, 73%) after chronic ART for four or more years. The average magnitude of HR reduction during ART on-time in all patients was 2.4 ± 3.2 bpm at the chronically programmed VNS pulse parameter settings.

**Conclusion:**

Autonomic responsiveness to VNS persists in patients with HFrEF who received chronic ART for up to 5 years as a supplement to GDMT. This suggests that the effects of ART on autonomic engagement and cardiac control remain durable over time.

**Clinical Trial Registration:**

[ClinicalTrials.gov], identifier [#NCT01823887, CTRI registration #CTRI/2012/05/002681].

## Introduction

Chronic heart failure (HF) is characterized by autonomic dysfunction, including excessive sympathetic activation and concomitant parasympathetic withdrawal (Eckberg et al., 1971; Brunner-La Rocca et al., 2001). This autonomic imbalance is associated with cardiovascular dysregulation, worsening HF, and increased risk of mortality independent of ejection fraction (EF) and ventricular arrhythmias (La Rovere et al., 1998; Nolan et al., 1998).

The ANTHEM-HF Study (ClinicalTrials.gov #NCT01823887) was a multicenter, open-label feasibility study that evaluated open-loop autonomic regulation therapy (ART) utilizing cervical vagus nerve stimulation (VNS) and found that ART was associated with sustained improvement at 6 months after VNS titration in heart rate (HR), HR variability, left ventricular (LV) function, HF symptoms, and quality of life in patients with chronic, stable, symptomatic HF with reduced EF (HFrEF) (Premchand et al., 2014, 2016). An analysis of electrocardiogram (ECG) recordings from ANTHEM-HF patients showed that instantaneous HR change during therapeutic levels of VNS can be used as a biomarker of autonomic engagement and cardiac control (Nearing et al., 2016). Autonomic engagement was reflected in distinct, observable changes in HR dynamics that occurred during VNS (Libbus et al., 2017).

Whether similar biomarker changes are observable after longer periods of ART is unknown. Therefore, we sought to evaluate changes in HR dynamics after 4 or more years of continuous ART for patients with HFrEF. We hypothesize that vagal response to ART, as reflected in acute HR response to vagus stimulation during the VNS duty cycle, is maintained after prolonged chronic VNS.

## Materials and Methods

### Study Design

**FIGURE 1:**
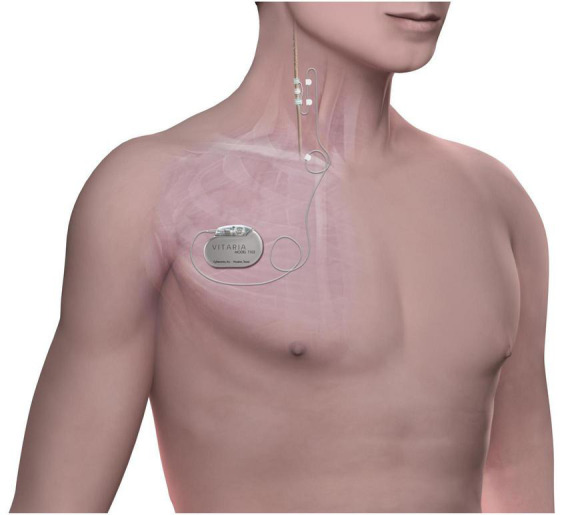
The stimulation system consists of an implantable pulse generator connected to a lead placed on the right cervical vagus nerve. Chronic cyclic stimulation is delivered throughout the follow-up period.

The ANTHEM-HF study design has been previously published (DiCarlo et al., 2013). Briefly, the ANTHEM-HF study enrolled 60 subjects in NYHA class II-III HF with LVEF ≤40% who were on guideline-directed medical therapy (GDMT). Subjects received VNS Therapy System implantation (Demipulse Model 103 pulse generator and PerenniaFLEX Model 304 lead, Cyberonics, Houston, TX, United States, Figure 1) with lead placement on the cervical vagus nerve. Stimulation parameters were systematically adjusted over a 10-week titration period to a pulse width of 250 μs and a pulse frequency of 10 Hz in all patients while gradually increasing output current. The mean output current at the end of titration was 2.0 ± 0.6 mA, and this stimulation was maintained throughout the follow-up period. Subjects who consented to extended follow-up received cardiovascular function and HF symptom assessments every 3 months until 42 months post-titration.

At the end of the extended follow-up period, all active patients with lead placement on the right vagus nerve were asked to participate in an additional follow-up visit to assess autonomic engagement. At the time of the follow-up visit, the 15-patient cohort who agreed to participate in this study had been chronically stimulated for 4.7 ± 0.3 years (range: 4.0–5.0 years). Mean stimulation parameters were a current amplitude of 2.2 ± 0.6 mA, frequency of 5 Hz, pulse width of 250 μs, and duty cycle of 14 s ON/66 s OFF.

### Autonomic Engagement Assessment

Assessment of autonomic engagement using sequential heart interval patterns during VNS with signal processing algorithms in patients with HF has been previously reported (Nearing et al., 2016). The instantaneous, transient HR changes in response to cycles of VNS are subtle, and can be either a small (0–3%) HR increase or decrease based on the intensity of the stimulation and its relationship to the neural fulcrum (Ardell et al., 2017). The neural fulcrum is defined as the operating point where a null HR response is reproducibly evoked during stimulation on-time, between the mild HR elevation observed with low-intensity VNS and the progressive HR reduction observed with higher-intensity ART (Ardell et al., 2017).

Autonomic engagement was assessed by measuring the instantaneous HR response to vagal stimulation during the VNS duty cycle, and the pattern of HR dynamics during the stimulation off-time compared to the on-time. The titration assessment system (TASys) consisted of a data acquisition and display system connected to lead I of the patient’s ECG. The Electrocardiogram signal was analyzed in real time to detect ventricular depolarization (R-wave). HR analysis and beat detection was previously validated against a publicly available ECG database (PhysioNet, MIT Laboratory for Computational Physiology) and found to be more than 99% accurate.

All ECG recordings were taken with the patient at rest, and efforts were made to maintain a stable HR during the entire recording. Recordings were made with patients lying on their back, with their arms in a relaxed position at their side. Electrodes were placed on the patient’s wrists, and a stable ECG was recorded from all patients. Unnecessary noise, conversation, and foot traffic was kept to a minimum, and the lighting in the room was kept as low and calming as possible.

**FIGURE 2:**
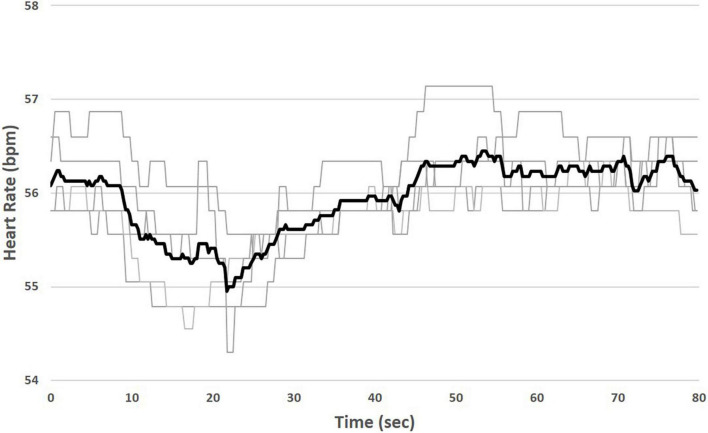
Measurement of instantaneous heart rate (HR) response to stimulation. Median HR is measured during ART on-time (shaded) and compared to median HR during an equivalent preceding ART off-time. Variability due to respiration and environmental effects during individual stimulation cycles (light traces) is minimized by averaging over multiple cycles (bold trace).

The chronic therapy delivered by the VNS Therapy System consists of intermittent, cyclic stimulation with a programmable duty cycle. In this study, the duty cycle was fixed at 14 s ON, 66 s OFF. TASys recordings were synchronized to the patient’s duty cycle, which allowed the system to determine in real time which cardiac beats occurred during ART on-time and which beats occurred during ART off-time. The TASys measured the median HR during ART on-time and compared it to the median HR during an equivalent preceding ART off-time period. This difference was calculated for each stimulation on/off cycle and averaged over a minimum of 10 consecutive cycles to minimize the influence of respiration and environmental effects on HR dynamics. Averaging over multiple cycles minimizes the variability of the HR measurement and accentuates the acute HR response (Figure 2).

## Results

### Patient Characteristics

The baseline characteristics of the 15 patients who consented to participate in this autonomic engagement assessment are summarized in Table 1 and compared with all 60 ANTHEM-HF patients. There were no significant differences between the two populations. At baseline (ANTHEM-HF enrollment), sub-study patients were either NYHA class II (53%) or class III (47%), with an average LVEF of 30.9 ± 7.1%. The average LVESV and LVEDV were 96 ± 30 mL and 142 ± 37 mL, respectively. Patients received optimum pharmacological therapy for HF, with 100% receiving β-blocker therapy and 84% receiving ACE inhibitor or ARB therapy.

**TABLE 1:** Baseline Demographics.

	ANTHEM-HF Patients (*n* = 60)	Study patients (*n* = 15)
Age	51.5 ± 12.2	52.7 ± 14.2
Gender (% Male)	87	93
Height (cm)	162 ± 6.9	162 ± 5.4
Weight (kg)	63.0 ± 10.9	61.7 ± 12.2
Heart rate (bpm)	77.6 ± 10.2	73.7 ± 11.0
Baseline NYHA class (II/III)	34/26	8/7
Baseline LVEF (%)	32.4 ± 7.2	30.9 ± 7.1
Baseline LVEDV (mL)	160 ± 50	142 ± 37
Baseline LVESV (mL)	108 ± 40	96 ± 30

### Autonomic Engagement Assessment

**FIGURE 3:**
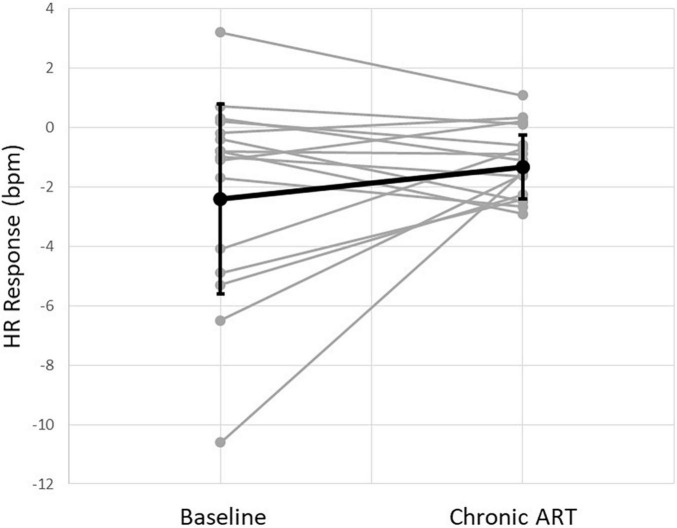
Instantaneous heart rate (HR) response to stimulation from all patients at baseline and at the time of chronic follow-up. Individual patients (gray) and an average of all patients (black) are shown.

Electrocardiogram records containing at least 10 on/off cycles were successfully recorded in all 15 patients. Autonomic engagement, defined as a measured HR reduction during ART on-time as compared to ART off-time, was calculated and averaged across all stimulation cycles for each record. Acute HR reduction was observed in a majority of patients (11 of 15, 73%) at the patient’s chronically programmed stimulation settings (2.2 ± 0.6 mA current amplitude, 250 μs pulse width, 5 Hz stimulation frequency). The average magnitude of HR reduction during ART on-time at the chronically programmed settings was 2.4 ± 3.2 bpm. This represents a substantial maintenance of the HR response magnitude that was observed at baseline (Figure 3).

## Discussion

Autonomic engagement as demonstrated by an acute reduction in HR during VNS was measurable in a majority of patients with HFrEF who had been receiving ART chronically for up to 5 years. This suggests that the effect of ART on autonomic engagement and cardiac control is durable over time.

Other recent randomized studies of VNS in HF were unable to demonstrate acute HR changes or chronic changes in HR and HR variability at 6 months or longer in response to VNS (Zannad et al., 2015a,b; Gold et al., 2016). This appears to have been related to inability to achieve an adequate VNS intensity utilizing the parameters that were selected in these studies, INOVATE-HF and NECTAR-HF, and resulted in symptomatic and functional changes that were significantly less than occurred in ANTHEM-HF.

In the ANTHEM-HF study, chronic ART was shown to be safe, tolerable, and associated with persistent improvement in cardiac function, HF symptoms, and exercise tolerance after 12 and 42 months of chronic therapy (Premchand et al., 2016; Sharma et al., 2020). The current analysis examined a cohort of patients who agreed to participate in this study after receiving chronic therapy for almost 5 years, and found that the majority of these patients exhibited an acute HR reduction in response to VNS that was similar in magnitude to the HR reduction originally observed at end-titration. These results suggest that chronic vagal response to VNS does not exhibit any tachyphylaxis but persists over time.

While demonstration of acute autonomic engagement during VNS may have potential as a tool for guiding adequate delivery of ART, the results of this analysis should be considered with caution. No gold standard for measurement of autonomic engagement biomarker has yet been established. This analysis examined a subset of the ANTHEM-HF study patient population. In contrast to the findings observed at end-titration in the full study population, the analysis of this patient subset did not demonstrate a HR response that was dependent on stimulation amplitude; this may have been related to the relatively small sample size that was evaluated.

## Conclusion

Appropriate levels of autonomic engagement are discernible using acute, transient HR changes in response to VNS as a biomarker. Autonomic engagement persists in patients with HFrEF who received chronic ART for up to 5 years as a supplement to GDMT. These findings suggest that the vagal response to ART remains durable over time, and TASys may be potentially useful for guiding VNS titration to provide adequate dosing of ART.

## Data Availability Statement

The raw data supporting the conclusions of this article will be made available by the authors, without undue reservation.

## Ethics Statement

The studies involving human participants were reviewed and approved by the Institutional Ethics Committee, Care Foundation, Care Hospital; Institutional Ethics Committee, Madras Medical Mission; KIMS Intuitional Ethics Committee; Institutional Ethics Committee, Yashoda Group of Hospital Hyderabad; Sanjivani Hospital Ethics Committee; Vintage Institutional Ethics Committee. The patients/participants provided their written informed consent to participate in this study.

## Author Contributions

IL contributed to collecting and analyzing data and drafting the manuscript. RP, KS, SM, and RM contributed to data collection. BA, BK, and IA contributed to reviewing the data and drafting the manuscript. All authors contributed to the article and approved the submitted version.

## Conflict of Interest

The authors declare that this study received funding from LivaNova. The funder had the following involvement in the study: study design, data collection and analysis, and manuscript preparation.

## Publisher’s Note

All claims expressed in this article are solely those of the authors and do not necessarily represent those of their affiliated organizations, or those of the publisher, the editors and the reviewers. Any product that may be evaluated in this article, or claim that may be made by its manufacturer, is not guaranteed or endorsed by the publisher.

## References

[B1] Ardell J. L. Nier H. Hammer M. Southerland E. M. Ardell C. L. Beaumont E. (2017). Defining the neural fulcrum for chronic vagus nerve stimulation: implications for integrated cardiac control. *J. Physiol*. 595 6887–6903. 10.1113/JP274678 28862330 PMC5685838

[B2] Brunner-La Rocca H. P. E. M. Jennings G. L. Kaye D. M. (2001). Effects of cardiac sympathetic nervous activity on mode of death in congestive heart failure. *Eur. Heart J*. 22 1136–1143. 10.1053/euhj.2000.2407 11428854

[B3] DiCarlo L. Libbus I. Amurthur B. Kenknight B. H. Anand I. S. (2013). Autonomic regulation therapy for the improvement of left ventricular function and heart failure symptoms: the ANTHEM-HF study. *J. Card Fail*. 19 655–660. 10.1016/j.cardfail.2013.07.002 24054343

[B4] Eckberg D. L. Drabinsky M. Braunwald E. (1971). Defective cardiac parasympathetic control in patients with heart disease. *N. Engl. J. Med*. 285 877–883. 10.1056/NEJM197110142851602 4398792

[B5] Gold M. Berman B. J. Borggrefe M. Spencer H. Randall C. Mann D. L. (2016). The Effect of Vagus Nerve Stimulation in Heart Failure: primary Results of the INcrease of VAgal TonE in Chronic Heart Failure (INOVATE-HF) Trial. *Am. Coll. Cardiol. Sci. Sess.* 68 149–158. 10.1016/j.ahj.2012.03.021 27058909

[B6] La Rovere M. T. Bigger J. T. Jr. Marcus F. I. Mortara A. Schwartz P. J. (1998). Baroreflex sensitivity and heart-rate variability in prediction of total cardiac mortality after myocardial infarction. ATRAMI (Autonomic Tone and Reflexes After Myocardial Infarction) Investigators. *Lancet*. 351 478–484. 10.1016/s0140-6736(97)11144-8 9482439

[B7] Libbus I. Nearing B. D. Amurthur B. KenKnight B. H. Verrier R. L. (2017). Quantitative evaluation of heartbeat interval time series using Poincare analysis reveals distinct patterns of heart rate dynamics during cycles of vagus nerve stimulation in patients with heart failure. *J. Electrocardiol*. 50 898–903. 10.1016/j.jelectrocard.2017.06.007 28625397

[B8] Nearing B. D. Libbus I. Amurthur B. Kenknight B. H. Verrier R. L. (2016). Acute Autonomic Engagement Assessed by Heart Rate Dynamics During Vagus Nerve Stimulation in Patients With Heart Failure in the ANTHEM-HF Trial. *J. Cardiovasc. Electrophysiol*. 27 1072–1077. 10.1111/jce.13017 27221316

[B9] Nolan J. Batin P. D. Andrews R. Lindsay S. J. Brooksby P. Mullen M. (1998). Prospective study of heart rate variability and mortality in chronic heart failure: results of the United Kingdom heart failure evaluation and assessment of risk trial (UK-heart). *Circulation*. 98 1510–1516. 10.1161/01.cir.98.15.1510 9769304

[B10] Premchand R. K. Sharma K. Mittal S. Monteiro R. Dixit S. Libbus I. (2014). Autonomic regulation therapy *via* left or right cervical vagus nerve stimulation in patients with chronic heart failure: results of the ANTHEM-HF trial. *J. Card Fail*. 20 808–816. 10.1016/j.cardfail.2014.08.009 25187002

[B11] Premchand R. K. Sharma K. Mittal S. Monteiro R. Dixit S. Libbus I. (2016). Extended follow-up of patients with heart failure receiving autonomic regulation therapy in the ANTHEM-HF study. *J. Card Fail*. 22 639–642. 10.1016/j.cardfail.2015.11.002 26576716

[B12] Sharma K. Premchand R. K. Mittal S. Monteiro R. Libbus I. DiCarlo L. A. (2020). Long-term Follow-Up of Patients with Heart Failure and Reduced Ejection Fraction Receiving Autonomic Regulation Therapy in the ANTHEM-HF Pilot Study. *Int. J. Cardiol.* 323 175–178. 10.1016/j.ijcard.2020.09.072 33038408

[B13] Zannad F. De Ferrari G. M. Tuinenburg A. E. Wright D. Brugada J. Butter C. (2015a). Chronic vagal stimulation for the treatment of low ejection fraction heart failure: results of the NEural Cardiac TherApy foR Heart Failure (NECTAR-HF) randomized controlled trial. *Eur. Heart J*. 36 425–433. 10.1093/eurheartj/ehu345 25176942 PMC4328197

[B14] Zannad F. De Ferrari G. M. Tuinenburg A. E. Wright D. Brugada J. Butter C. (2015b). Long Term Safety and Efficacy Results of the NEural Cardiac TherApy foR Heart Failure (NECTAR-HF) Trial. *J. Card Fail*. 21:936. 10.1016/j.ijcard.2017.06.036 28663046

